# Mind and Health

**Published:** 1855-05

**Authors:** 


### MIND AND HEALTH.

Women, the world over, are inquisitive, very; as largely so out of their own sphere as in it. A lady correspondent desires to know what the diary of Mr. and Mrs. Sparrowgrass, in a previous number, had to do with health? If courtesy had not forbidden, I might have referred her to a Down Easter, on his way to Boston, who is said to have been questioned by an acquaintance thus:—

*Friend.*—Why Eb! where are you going to-day?

*Ebenezer.*—To Boston.

*Friend.*—Why, what upon earth takes you there this time of year?

*Eb.*—To get my pension.

*Friend.*—Pension! why, for what?

*Eb.*—For services done the country.

*Friend.*—What! and how much? *Does it pay?*

*Eb.*—Yes; well. I get two cents for minding my own business, and two cents for letting other people's alone!

But I did not refer my fair inquisitor to Eb; and more pressing matters prevented a reply. I have thought since, that perhaps other readers may have had the same inquiry suggested. It might be well to reply here, once for all.

1. The first object in determining me tc found this Journal, was personal profit.

2. The second object was personal pleasure.
3. The third object was public good.

As far as I know, I have not as yet failed in either of these three respects, and trust that I will not. The second object answers the inquiry. But in connection, I wish to make a statement, in reference to which, there is a very great oversight in the public mind; and that is, that when the subject of health is concerned, we instinctively think of physic and physicians, of surfeits, and exposures, of dieting, and starvation, of cramps and pains and all that; but of the states of the mind in their bearing on health and disease, we hardly ever make a note. If a man dies of consumption, or "sweet seventeen" pales and pines away and dies, it does not therefore follow that exposure to wind and rain, or thin stockings, or wet feet, have done the injury. If another dies of inflammation of the brain, we are not to conclude necessarily, that intense devotion to some literary or scientific subject, has worked the mischief. If still another looks well in the face, but has no relish for food, has no sweet sleep at night, but dreams and tosses and tumbles by the hour; and if ever a smile lights up his countenance, it is as fitful and as transient, as a spark from the smoke-pipe of a locomotive; these manifestations are not as a matter of course the effect of some bodily disease, or functional derangement; but in cases least thought of, the malady is in the mind—a mind working so intensely as to eat out all the nervous supplies, not leaving enough to carry on the bodily machinery. A case of this kind has recently occurred in Washington city, in reference to a gentleman whose indomitable perseverance in following out his plans, when water and winds and fire and man, seemed combined to thwart them, and who for weary months of discouragement and long years of baffled purposes, right nobly has stood up, and breasted all, but in the last conflict he died, March 5th, 1855, at his residence, on Capitol Hill, Washington city, and as is believed, from the effects which ill treatment by some of the officers at Washington, had on his mind. The highest authority inquires—*A wounded spirit, who can bear?* It was Robert Mills, civil engineer, and planner of the national monument. From similar causes, many a noble heart has perished before, whose organization was too highly strung to meet the rude buffetings of a world like this. Thus it is, that I address myself often, in the conduct of this Journal, to the passions, to the sensibilities, to the social conduct, as a means of health, and as a cause of disease. Laughing makes the body fat, is a familiar and a truthful saying; therefore what promotes innocent and instructive laughter, promotes health; and if any body can read Mr. and Mrs Sparrowgrass's experience in living in the country, and not laugh until the tears run down the cheeks, as freely as if a whole onion had been squeezed into each eye, it is rather more than I can do!

Whatever promotes a comfortable and harmless state of the mind, promotes health. If I tell my readers what politeness is, and how they may become so, and it is practiced, I thus am found in the legitimate conduct of a health journal. Who does not know that a single courteous act, or even word, will sometimes break up in an instant, a reverie of sadness, and place a gladness, where, but an instant before, there was gloom. And any observant reader may, in his own experience, harrow up instances, full numerous even in a short life, where an act of thoughtless or unexpected, or undeserved rudeness, has caused a tempest of feeling which hours and days have failed to allay—aye, half a century sometimes, a fruitful source of fresh resentment whenever thought of, for the whole of after life—long after the churl or boor who excited it, has gone to his grave! In the same way, if I teach young men to be punctual, and thus save to others the fretfulness of disappointment; if I teach them to be methodical, and by having a place for every thing and every thing in its place, save them from fits of passion, by not finding it in its place on emergency; if I council them not to go in debt in early life, beyond what they have ample and certain means to pay, without a sacrifice, and thus save them from that wasting solicitude which has destroyed many a noble minded merchant; if I say, by these and other means, I promote politeness, punctuality, honorable dealing and other mental and moral virtues, I thus am promoting human happiness and necessarily human health. For morals, virtue, religion, health, all react on one another.

Southey was a man of method and of system. Although he effected so much, his friend Wordsworth said of him he never found him in a hurry, yet he accomplished an amount of labor, with such little apparent effort, as to be almost incredible. General Washington had such a special arrangement of his time at Mount Vernon, after retiring from the presidency, that he had his kindling wood brought in over night, so that he not only need not disturb the servants unnecessarily early, but that by kindling his own fires, his mind might not be ruffled in the early part of the day, to give its tinge to subsequent duties, and more, that he might not lose time in waiting for a servant, who might oversleep himself, or might fail occasionally to "hit it," in the way of kindling; thus wisely avoiding the way of temptation for himself, and sparing his servants, showing at once his *method*, his kindness of heart and his industry. How many an hour is wasted, no one can tell, by the young especially, in waiting in bed for the servant to come and kindle the fire of a cold morning, and what angry words and more angry thoughts are engendered every day for the want of *method*, in this little domestic item.

